# Eye-Resolvable Surface-Plasmon-Enhanced Fluorescence Temperature Sensor

**DOI:** 10.3390/nano12224019

**Published:** 2022-11-16

**Authors:** Luping Tang, Yangyang Zhang, Chen Liao, Longbing He, Xing Wu, Yiwei Liu, Litao Sun

**Affiliations:** 1 College of Mechanical and Electrical Engineering, Nanjing Forestry University, Nanjing 210037, China; 2 College of Electronic and Optical Engineering & College of Flexible Electronics (Future Technology), Nanjing University of Posts and Telecommunications, Nanjing 210023, China; 3 SEU-FEI Nano-Pico Center, Key Lab of MEMS of Ministry of Education, Southeast University, Nanjing 210096, China; 4 Shanghai Key Laboratory of Multidimensional Information Processing, East China Normal University, Shanghai 200241, China

**Keywords:** temperature sensor, fluorescence, Ag@SiO_2_@CdS/ZnS composite nanoparticle, peak intensity, peak wavelength

## Abstract

Temperature sensors are widely used in important fields such as daily home, medical care, and aerospace as a commonly used device for measuring temperature. Traditional temperature sensors such as thermocouples, thermal resistances, and infrared sensors are technically mature; however, they have limitations in the application environment, temperature measurement range, and temperature measurement accuracy. An eye-resolvable surface plasmon-enhanced fluorescence temperature sensor based on dual-emission Ag@SiO_2_@CdS/ZnS composite nanoparticle film with multiple-parameter detectable signals and high response sensitivity was proposed in this work. The temperature sensor’s x-chromaticity coordinate varied from 0.299 to 0.358 in the range of 77–297 K, while the y-chromaticity coordinate varied from 0.288 to 0.440, displaying eye-resolvable surface plasmon-enhanced fluorescence. The ratiometric response of two isolated photoluminescence (PL) peak-integrated areas located around 446 and 592 nm was found to be significantly temperature dependent, with a thermal sensitivity of 1.4% K^−1^, which can be used as an additional parameter to measure the precise temperature. Furthermore, the surface state emission peak intensity was linearly related to temperature, with a correlation index Adj. R-Square of 99.8%. Multiple independent temperature estimates can help with self-calibration and improve the measurement accuracy. Our findings show that the designed sensors can detect low temperatures while maintaining stability and reproducibility.

## 1. Introduction

Temperature sensor technology is widely used in aerospace, aviation, navigation, and other technical fields as well as in people’s daily work and lives [1,2]. Traditional temperature sensors such as thermocouples, thermal resistances, and infrared sensors are technically mature; however, they have limitations in the application environment, temperature measurement range, and temperature measurement accuracy. At low temperatures close to liquid nitrogen, conventional thermocouple and resistance temperature sensors would self-heat, making it difficult to accurately measure low temperatures [3]. Because of their tunable particle size and composition as well as their high luminous efficiency, semiconductor quantum dots (QDs) have recently become widely used in lighting-emitting diodes, solar cells, temperature sensors, and optically pumped lasers [4,5,6]. Luminescence thermometry is a general optical technique used to measure the local temperature by using the variation in the luminescence properties of an indicator or probe (emission intensity and wavelength) with temperature [7,8]. As a result, it is critical to investigate the optical properties of QDs as a function of temperature. Temperature affects the band gap and luminescence properties of semiconductors such as CdS [9], CdSe [10], CdTe [11], ZnSe [12], PbSe [13], and Ag_2_Se [14,15]. The surface states of single CdS nanowires rapidly quench at varying rates as the temperature increases, leaving the band-edge photoluminescence (PL) that dominates at room temperature [16]. CdS cores and CdS/CdZnS-ZnCdSe/ZnSe showed a broadening of the full width at half maximum and a red shift of the PL peaks, whereas CdS/ZnSe showed a blue shift of the PL peak at temperatures ranging from 10 to 300 K [17].

Compared to general organic fluorophores such as luminescent coordination complexes, inorganic phosphors, and organic dyes, QDs used as optical temperature probes have distinct advantages such as a broad excitation peak, a narrow emission peak, high fluorescence efficiency, and photobleaching resistance [18,19]. For example, CdSe QD wavelength shifts were used for local temperature measurements, and the spectral shift of a single QD was 0.1 nm/°C [20]. In the temperature range of 23–80 °C, CdTe QD-layered double hydroxide ultrathin films exhibited remarkable and reversible transitions (emission intensity and wavelength) [21]. However, several issues such as a single detectable signal, insufficient response sensitivity, poor thermal stability, and a limited temperature response range, remain unresolved in the application of QD-based temperature sensors. Furthermore, a human eye visible QD-based fluorescent temperature sensor is yet to be realized. As a result, the question of how to fabricate temperature sensors based on QDs with eye-resolvable fluorescence, multi-parameter signals, reproducibility, and stability remains unanswered.

CdS QDs are a valuable semiconductor nanomaterial with excellent optoelectronic properties. To achieve more stable blue light emission, one or more layers of semiconductor materials (such as ZnS) with a large band gap are typically passivated on the surface of CdS QDs to increase the coordination of surface atoms and block excitons in the CdS QDs from interacting with those in the surrounding environment, thereby improving the stability and fluorescence efficiency of CdS QDs. Because of their unique surface plasmon resonance properties, metal nanostructures have recently become a research hotspot [22,23,24]. Metal nanostructures have been shown in studies to improve the signal intensity and signal-to-noise ratio of QD-based detection systems by increasing the fluorescence emission intensity of QDs via the localized surface plasmon resonance effect [25,26]. According to our previous study [27], Ag@SiO_2_@CdS/ZnS composite nanoparticles with different thicknesses of SiO_2_ shell (2–30 nm) were prepared to modify the emission from trap-rich CdS/ZnS QDs by adjusting the distance between Ag nanoparticles and the QDs. Due to the strong plasmon coupling between the Ag nanoparticles and CdS/ZnS QDs, we could achieve optimal enhancements of the band-edge emission (BEE, four times) and surface state emission (SSE, 17 times) simultaneously with a SiO_2_ shell thickness of 10 nm. Multiple approaches to controlling the light emission of QDs are enabled by a strongly interacting metal-QD plasmonic coupled structure for applications in new QD light-emitting diodes, photovoltaic cells, biomarker detectors, and novel light sources [28].

This paper describes the development of an eye-resolvable surface plasmon-enhanced fluorescence temperature sensor based on a dual-emission Ag@SiO_2_@CdS/ZnS composite nanoparticle film. The PL properties of Ag@SiO_2_@CdS/ZnS composite nanoparticles were measured using the temperature-dependent steady-state and transient fluorescence from 77 to 297 K. The temperature sensor’s corresponding Commission Internationale del’Èclairage (CIE) chromaticity coordinates varied noticeably with temperature, displaying different eye-resolvable surface plasmon-enhanced fluorescence. The temperature sensitivity of the Ag@SiO_2_@CdS/ZnS composite nanoparticle film sensor was 1.4% K^−1^, higher than the temperature sensitivity of most previously reported QD-based temperature sensors. Furthermore, various PL-spectrum parameters such as peak intensity and peak wavelength were investigated as a function of temperature to aid self-calibration and improve the temperature detection accuracy. This work presents a simple, eye-resolvable, and low-temperature detectable approach for the fabrication of fluorescence temperature sensors as well as promising applications in visual detection.

## 2. Materials and Methods

### 2.1. Preparation of a Fluorescence Temperature Sensor

According to a previous study [27], Ag@SiO_2_@CdS/ZnS composite nanoparticles were synthesized. First, Ag nanoparticles were synthesized using the standard polyol method, and then the SiO_2_ shell was deposited using the Stöber method. Here, 5 μL TEOS was used to control the thickness of the SiO_2_ shell, which was about 10 nm. The reaction solution was then activated at 12,000 rpm after 10 μL (3-aminopropyl) triethoxylsiane (APS) was added to 2-mL Ag/SiO_2_ solution stirred at room temperature for 8 h. Finally, a small amount of water-soluble CdS/ZnS QDs were added to the ethanol solution of the aminated Ag@SiO_2_ nanoparticles and stirred for 1–2 h at room temperature. The film was prepared by spin coating Ag@SiO_2_@CdS/ZnS composite solutions onto a quartz substrate at 4500 rpm for 60 s using a Laurell WS-650MZ-23NPP adjustable speed homogenizer. The spun-coated Ag@SiO_2_@CdS/ZnS composite nanoparticles were placed in a vacuum liquid nitrogen cryostat within a temperature range of 77 to 297 K. The Ag@SiO_2_@CdS/ZnS composite nanoparticle film was spin-coated with a layer of polysilazane, which converts to a layer of silicon dioxide in the atmosphere to encapsulate the device.

### 2.2. Characterization Methods

Both low resolution and high-resolution transmission electron microscope (TEM) images were measured by Tecnai G20 of the FEI Company, with an acceleration voltage of 200 kV. The sample is dropped on the ultra-thin micro grid copper mesh covered with ultra-thin carbon film, and can be measured after the solvent evaporates. The atomic force microscope image is measured by a Multimode-8-AM atomic force microscope by Banker Nano. The absorption spectra were measured using a UV–Vis NIR spectrophotometer (UV-3600, Shimadzu). The temperature-variable measuring device of Edinburgh’s F900 steady-state/transient fluorescence spectrometer was used, and liquid nitrogen was used as the cooling material. The PL spectra and time-resolved PL decay spectra of all samples were acquired by a fluorescence spectrometer (F900, Edinburgh instrument) under excitation at 375 nm. When measuring the steady-state PL spectrum, the power density of the exciting laser radiation was 2 W/cm^2^. When measuring the time-resolved PL decay spectrum, no more than one emission photon was generated under 1000 pulse pumping through continuous attenuator adjustment. The PL spectrum and lifetime of the Ag@SiO_2_@CdS/ZnS composite nanoparticles were measured under variable temperature in the experiment. The temperature options are as follows: 77, 87, 97, 107, 117, 132, 147, 162, 177, 192, 207, 222, 237, 257, 277, and 297 K. At each temperature point, we measured the PL spectrum and the lifetime decay curve separately. To reduce the experimental error in the measurement results, 30 min was added after each stable temperature point to ensure that the difference between the temperature in the cryostat and the set temperature was as small as possible.

## 3. Results and Discussion

**Figure 1:**
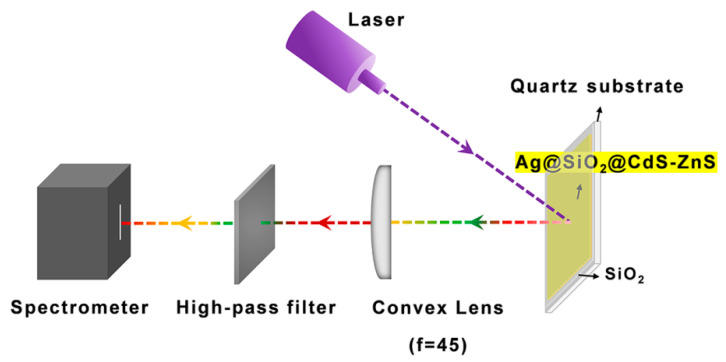
Fluorescence temperature sensor system based on a Ag@SiO_2_@CdS/ZnS composite nanoparticle film.

Figure 1 depicts the structure of a temperature sensor device with a broad measurement range based on the photo-induced fluorescence temperature change characteristics of an Ag@SiO_2_@CdS/ZnS composite nanoparticle film. The Ag@SiO_2_@CdS/ZnS composite nanoparticles were created using a multi-step procedure that included the fabrication of Ag nanoparticles, the deposition of SiO_2_ shell, the decoration of the SiO_2_ surface with –NH_2_ groups, and finally, the self-assembly of water-soluble CdS-ZnS on the particle surface, as detailed in the experimental section. Spin-coating was used to coat the prepared Ag@SiO_2_@CdS/ZnS composite nanoparticles on a quartz wafer, which was then placed in a vacuum liquid nitrogen cryostat with a temperature range of 77 to 297 K. The PL spectrum and lifetime of a Ag@SiO_2_@CdS/ZnS composite nanoparticle film were measured under variable temperature conditions in the experiment.

**Figure 2:**
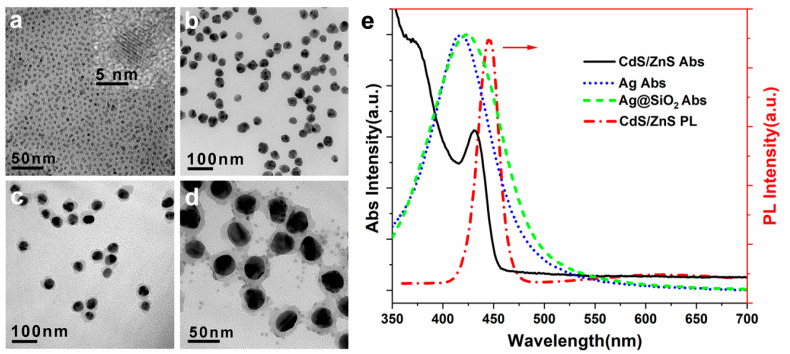
TEM images of the CdS/ZnS core/shell QDs (**a**), Ag nanoparticles (**b**), Ag@SiO_2_ core/shell nanoparticles with SiO_2_ shell thickness of about 10 nm (**c**), and Ag@SiO_2_@CdS/ZnS composite nanoparticles (**d**). (**e**) Absorption (Abs) spectra of Ag nanoparticles, Ag@SiO_2_ nanoparticles, CdS/ZnS QDs, and photoluminescence (PL) spectrum of CdS/ZnS QDs, respectively.

TEM images of CdS/ZnS core-shell QDs with an average size of 5.7 nm are shown in Figure 2a. The lattice spacing is clearly visible in the high-resolution transmission electron microscopy images (Figure 2a inset), indicating that the QDs are highly crystalline. Figure 2e shows the UV–Visible absorption and PL spectra of CdS/ZnS QDs. The absorption maximum of CdS/ZnS QDs was 431 nm. In addition to the first exciton absorption peak, the absorption spectrum showed several other absorption peaks, demonstrating that these CdS QDs had a narrow particle size distribution. The fluorescence spectrum showed that, in addition to a strong eigenstate emission peak, CdS QDs had an emission peak with a larger half width in the long wavelength direction. A high number of dangling bonds resulted in a high number of surface defect states. When the CdS QDs are excited, a portion of the photogenerated carriers is captured by these defect states, resulting in surface state luminescence. The peak positions of the BEE and SSE PL were 446 nm and 592 nm, respectively. The TEM image shows that the Ag nanoparticles have a quasi-spherical morphology with an average size of 32 nm (Figure 2b), and the nanoparticle-localized surface plasmon resonance peak (LSPR) was found at 412 nm (Figure 2e). The TEM image of Ag@SiO_2_ core/shell nanoparticles with a SiO_2_ shell thickness of about 10 nm is shown in Figure 1c, and the LSPR peak red-shifted to 424 nm (Figure 1e). The CdS/ZnS QDs formed uniform monolayers on the Ag@SiO_2_ nanoparticles, as shown in Figure 2d. Moreover, the thickness of the Ag@SiO_2_@CdS/ZnS composite nanoparticle film measured by the step meter was about 150 nm. Figure S1 shows the atomic force microscopy (AFM) diagram of a representative Ag@SiO_2_@CdS/ZnS composite nanoparticle film. Within the measurement range of 100 µm^2^, the root mean square and peak-to-peak surface roughness were ~6 nm and ~55 nm, respectively (much less than the excitation and emission wavelengths), indicating that the film had a high-quality surface morphology and did not cause large scattering noise. The light-emitting layer (Ag@SiO_2_@CdS/ZnS composite nanoparticle film) was composed of pure and dense Ag@SiO_2_@CdS/ZnS composite nanoparticles, and thus the distribution of nanoparticles in the film was uniform. The encapsulated SiO_2_ layer was only covered on the surface of the light-emitting layer film.

**Figure 3:**
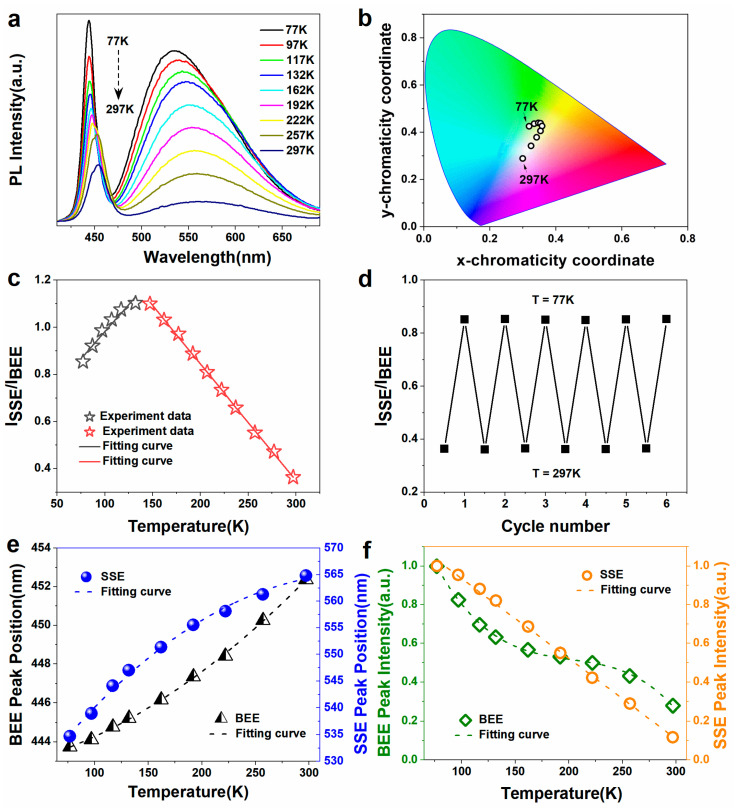
(**a**) The temperature-dependent PL spectra of the Ag@SiO_2_@CdS/ZnS composite nanoparticle film in the 77–297 K temperature range, respectively. (**b**) Temperature-dependent emission from the Ag@SiO_2_@CdS/ZnS composite nanoparticle film projected onto the Commission Internationale del’Èclairage (CIE) chromaticity diagram based on PL spectra shown in (**a**). (**c**) The ratio of the SSE and BEE integrated PL peak area. (**d**) Thermal stability of the integrated SSE and BEE PL peak area ratio over six cycles of heating and cooling between 77 and 297 K. (**e**) Temperature dependence of the PL emission peak shift of band-edge emission (BEE) (spheres) and surface state emission (SSE) (triangles), respectively. (**f**) Temperature dependence of the BEE (diamond) and SSE (circle) peak intensity, respectively.

Figure 3a depicts the temperature-dependent PL spectra of a Ag@SiO_2_@CdS/ZnS composite nanoparticle film recorded at temperatures ranging from 77 to 297 K. The PL spectra of a Ag@SiO_2_@CdS/ZnS composite nanoparticle film changed as the temperature changed. The BEE intensity of a Ag@SiO_2_@CdS/ZnS composite nanoparticle film decreased with increasing temperature, which was attributed to the thermally activated nonradiative defects. The SSE intensity of the Ag@SiO_2_@CdS/ZnS composite nanoparticle film decreased as the test temperature rose. It has been reported in the literature that the carriers of the intrinsic state will be transferred to the surface state (the trapping process of the surface trap state), increasing the luminescence intensity of the surface state [29]. Simultaneously, the effect of thermally activated nonradiative defects exists in this luminescence process, so the temperature-dependent change trend of the SSE intensity is determined by the competition of these two processes [9]. The quantum yield of the Ag@SiO_2_@CdS/ZnS composite nanoparticle film at 297 K and 77 K were about 17% and 90%, respectively.

The CIE chromaticity diagram depicts the trajectory of the (x, y) coordinates of the temperature-dependent dual-emission PL-spectrum from the Ag@SiO_2_@CdS/ZnS composite nanoparticle film (Figure 3b). The CIE chromaticity coordinates were (0.319, 0.425), (0.335, 0.436), (0.347, 0.440), (0.354, 0.438), (0.358, 0.426), (0.353, 0.406), (0.342, 0.379), (0.325, 0.342), (0.299, 0.288), which change dramatically with temperature. At 77 K, the ratio of the SSE and BEE emission intensities was 0.85, resulting in the effective green color. The BEE intensity decreased nearly half-fold at temperatures ranging from 77 to 162 K, while the SSE intensity decreased by about 30%. Because of these changes, the overall spectrally integrated PL intensity decreased by 30% and the emission color changed to yellow, as shown in Figure 3b. When the temperature was set to 297 K, the ratio of the SSE and BEE emission intensities was 0.36, and the CIE chromaticity diagram showed that the (x, y) coordinate of the emission light from the Ag@SiO_2_@CdS/ZnS composite nanoparticle film was located in the white area. Under different temperatures, the corresponding x-chromaticity coordinate of the as-prepared sensor was verified from 0.299 to 0.358, while the y-chromaticity coordinate was verified from 0.288 to 0.440, indicating that the temperature sensor displayed eye-resolvable surface plasmon-enhanced fluorescence. To demonstrate the function of Ag and SiO_2_ in plasmon-enhanced fluorescence and highlight why Ag@SiO_2_(10 nm)@CdS/ZnS composite nanoparticles were chosen to prepare the temperature sensor in this work, the CdS/ZnS nanoparticle film was also investigated. Figure S2a depicts the temperature-dependent PL spectra of a CdS/ZnS composite nanoparticle film recorded at temperatures ranging from 77 to 297 K. The CIE chromaticity diagram depicts the trajectory of (x, y) coordinates of the temperature-dependent PL-spectrum from the CdS/ZnS QD film (Figure S2b). Compared to Ag@SiO_2_@CdS/ZnS PCs, as the temperature increased, the SSE intensity changed very little (Figure S2a) and the corresponding range of color coordinate changes was also very narrow (Figure S2b). As shown in Figure S2b, the emission color range span was small, mainly distributed in the blue area. Moreover, the quantum yield of the CdS/ZnS QD film at 297 K was about 2%, indicating the PL signal of CdS/ZnS QDs was weak.

Temperature-dependent dual-emission PL spectroscopy allows for ratiometric measurements. As shown in Figure 3c, the ratiometric measurement result of the integrated PL peak area, $R=I_{\mathrm{SSE}}/I_{\mathrm{BEE}}$, showed two distinct regions. R is linearly dependent on temperature, with a slope of 0.00459 K^−1^ at low temperatures (77–130 K), and a slope of −0.00496 K^−1^ in the range of 130–297 K. Table 1 shows the linear function relationship parameters. The thermal sensitivity S is defined as $S=\frac{1\mathrm{dR}}{\mathrm{RdT}}$, where R is the integrated PL peak area ratio at a given temperature (T) [7,30]. The maximum sensitivity value for the Ag@SiO_2_@CdS/ZnS composite nanoparticle film was easily observed to be 1.4% K^−1^ measured at 297 K, which was higher than most of the previously reported temperature sensors based on semiconductor QDs (Table 2). In comparison to CdHgTe QDs@NaCl and CdTe QD-layered double hydroxide ultrathin film luminophores, the dual-emission peaks of the Ag@SiO_2_@CdS/ZnS composite nanoparticle film displayed two resolvable emission bands for different eye-resolvable surface plasmon-enhanced fluorescence. Furthermore, the dual emission with two different lifetimes (several nanoseconds for BEE and tens of nanoseconds for SSE) allows for the mutual calibration, which improves the temperature detection accuracy. The cycling experiment results are shown in Figure 3d; a minimal change in the PL peak intensities was observed during six consecutive cycles of repeated heating and cooling between 77 and 297 K, indicating its excellent reversibility and reliability as an optical sensor for thermometer applications. As a result, the integrated PL peak area ratio can be used as a further parameter to determine the precise temperature.

Furthermore, the relationship between the PL spectra parameters and temperature, which included the PL peak wavelength and peak intensity, was investigated. The PL peak wavelength clearly redshifted as the temperature increased, indicating that the energy gap shrinks with increasing temperature due to the lattice transformation state and exciton-phonon coupling [38,39]. Figure 3e depicts the relationships between the SSE and BEE peak wavelengths of the Ag@SiO_2_@CdS/ZnS composite nanoparticle film and temperature. A quadratic fitting polynomial can be used to represent the variation in the peak wavelength of the PL spectrum of the Ag@SiO_2_@CdS/ZnS composite nanoparticle film with temperature, and the quadratic function relationship parameters are shown in Table 3. The Adj. R-Square represents the quadratic fitting degree, and the closer it is to one, the better [1]. Clearly, the Adj. R-Square of the relationship between the BEE or SSE peak wavelength of the temperature sensor based on the Ag@SiO_2_@CdS/ZnS composite nanoparticle film and temperature was greater than 0.995, indicating that both had an apparent quadratic function relationship. The dotted line in Figure 3e represents the quadratic fitting relationship between the PL peak wavelength and temperature, as shown in Figure 3a. Because the PL peak wavelength can be obtained by monitoring the PL spectrum, the temperature value can be calculated using the quadratic fitting relationship between the PL peak wavelength of the QD-based sensor and temperature. The temperature dependence of the Ag@SiO_2_@CdS/ZnS composite nanoparticles synthesized in the same batch was noted to be relatively stable. As a result, after calibrating a fluorescence sensor from the same batch of QD-based sensors, the fluorescence temperature sensors can be used to detect temperature.

The PL spectra of the Ag@SiO_2_@CdS/ZnS composite nanoparticle film were strongly temperature dependent. Figure 3f shows the PL peak intensity of a Ag@SiO_2_@CdS/ZnS composite nanoparticle film as a function of temperature from 77 to 297 K. The PL intensity of the Ag@SiO_2_@CdS/ZnS composite nanoparticle film decreased rapidly with increasing temperature, as shown in Figure 3a, indicating the presence of many nonradiative traps on the nanoparticle surface, resulting in strong thermal quenching. At room temperature, PL from a relatively broad excitonic emission near the CdS/ZnS band-edge dominated the emission from the Ag@SiO_2_@CdS/ZnS composite nanoparticle film. However, at low temperatures, the nanoparticles’ internal structure generated two PL bands associated with an exciton emission near the CdS/ZnS band-edge and defect emission at lower energies. The normalized PL spectra intensity of the Ag@SiO_2_@CdS/ZnS composite nanoparticle film at different temperatures is displayed to visualize the variation in the PL peak intensity with temperature. The linear fit relationship between the SSE peak intensity and temperature is shown in Figure 3f. A cubic fitting polynomial can simply represent the variation in the peak intensity of the BEE PL spectrum of the Ag@SiO_2_@CdS/ZnS composite nanoparticle film with temperature. Table 4 displays the linear or cubic function relationship parameters. The results show that the SSE peak intensity had the best linear relationship with temperature increase among the PL spectral parameters, and the corresponding correlation index Adj. R-Square was 99.8%.

**Figure 4:**
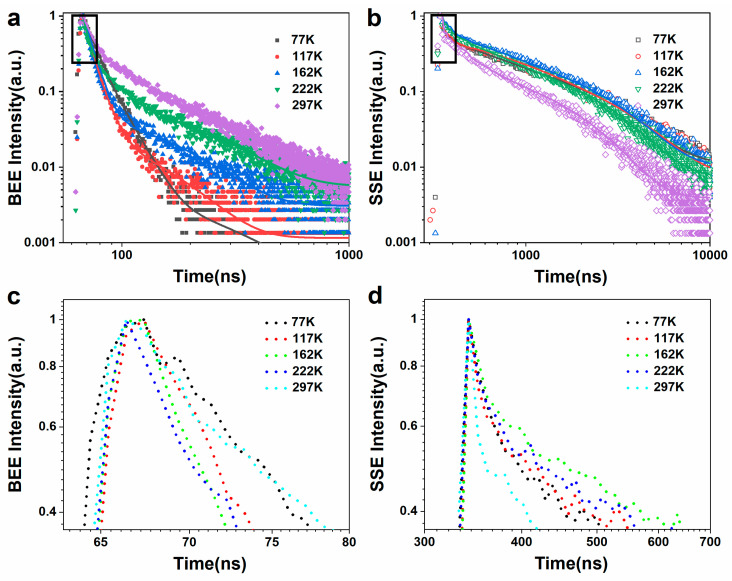
BEE (**a**) and SSE (**b**) fluorescence lifetime of the Ag@SiO_2_@CdS/ZnS composite nanoparticle film in the range of 77–297 K, respectively. (**c**,**d**) Zoom-in of the BEE and SSE decay curves at short times, respectively.

Figure 4a,b shows the BEE and SSE fluorescence lifetimes of a Ag@SiO_2_@CdS/ZnS composite nanoparticle film at various temperatures ranging from 77 to 297 K. The triple exponential mode fit all fluorescence relaxation curves well with $F\left(t\right)=A+B_{1}\exp\left(-t/\tau_{1}\right)+B_{2}\exp\left(-t/\tau_{2}\right)+B_{3}\exp\left(-t/\tau_{3}\right)$, where $\tau_{i}$ is the characteristic lifetime component, $B_{i}$ is the exponential term coefficient, and *A* is the additional background value, respectively. Average lifetime is calculated using $\tau =\left(B_{1}\tau_{1}^{2}+B_{2}\tau_{2}^{2}+B_{3}\tau_{3}^{2}\right)/\left(B_{1}\tau_{1}+B_{2}\tau_{2}+B_{3}\tau_{3}\right)$. The fluorescence lifetime decay curve in this experiment can be fitted by a triple exponential decay function, revealing three exciton recombination processes in nanocrystals: a fast decay process ($\tau_{1}$) with a time of several nanoseconds; a moderate decay process ($\tau_{2}$), with a time of 10 nanoseconds; and a slow decay process ($\tau_{3}$), with a time of close to one hundred nanoseconds. The fast decay process is thought to be caused by the direct recombination of internal excitons [40,41,42], whereas the moderate and slow decay processes are thought to be caused by the interaction of exciton states and surface states [43,44,45]. At room temperature, the BEE and SSE lifetimes of the Ag@SiO_2_@CdS/ZnS composite nanoparticle film were 5.8 ns and 6.8 ns, respectively, with the SSE lifetime being longer than the corresponding BEE lifetime due to the trapping and releasing effects of surface states. The fluorescence lifetimes of the SSE of the Ag@SiO_2_@CdS/ZnS composite nanoparticle film were 25.9 ns at 77 K and 6.8 ns at 297 K, with high-temperature decaying faster than low-temperature decaying (Figure 4b,d). Nonradiative decay is significantly enhanced at high temperatures, decreasing the luminous intensity [21,46]. Furthermore, it has the largest exponential term coefficient $B_{1}$ among $\tau_{i}$, so the average lifetime is primarily determined by $\tau_{1}$, exhibiting a lifetime of several to tens of nanoseconds, which can support a very fast device response, as shown in Figure 4c,d. As a result, the Ag@SiO_2_@CdS/ZnS composite nanoparticle film’s dual parameters (steady-state and transient fluorescence) will ensure its practical application.

## 4. Conclusions

A dual-emission Ag@SiO_2_@CdS/ZnS composite nanoparticle film was used to construct a fluorescence temperature sensor. The PL spectral intensity gradually decreased with increasing temperature in the temperature range of 77–297 K. Under different temperatures, the as-prepared sensor’s corresponding x-chromaticity coordinate varied from 0.299 to 0.358, while the y-chromaticity coordinate varied from 0.288 to 0.440, displaying eye-resolvable surface plasmon-enhanced fluorescence. The ratiometric response of the two isolated PL peak-integrated areas at around 446 and 592 nm was found to be significantly temperature dependent, with a thermal sensitivity of 1.4% K^−1^, which can be used as an additional parameter to measure the precise temperature. Furthermore, the temperature-dependent PL peak intensity and peak wavelength were investigated to facilitate self-calibration and improve the temperature detection accuracy. This work demonstrates the successful design and fabrication of a simple, low-temperature detectable and eye-resolvable fluorescent film based on the Ag@SiO_2_@CdS/ZnS composite nanoparticles as well as its promising application as a fluorescence temperature sensor.

## Figures and Tables

**Table 1:** Temperature dependence of the integrated SSE and BEE PL peak area ratio of the Ag@SiO_2_@CdS/ZnS composite nanoparticle film sensor.

Temperature Range (K)	Linear or Cubic Function Relation	Adj. R-Square
77–130	W=0.52293+0.00459T	0.94045
130–297	W=1.83571−0.00496T	0.9993

**Table 2:** Comparison of the previous reported temperature sensors based on semiconductor QDs.

Sensing Materials	Temperature Range (K)	Sensitivity (% K^−1^)	Ref.
Ag@SiO_2_@CdS/ZnS	77–297	1.4	In this work
CdSe QDs	82–280	0.69	[31]
Mn^2+^-doped ZnSe-ZnCdSe QDs	82–280	0.9	[32]
Mn^2+^-doped CdS-ZnS QDs	77–320	0.5	[33]
PbS/CdS giant QDs	150–373	1.13	[34]
PbS/CdS/CdSe QDs	180–300	1.22	[7]
CuInS_2_ QDs	140–340	1.0	[35]
Zn_1−x_Mn_x_Se/ZnS/CdS/ZnS QDs	373–673	0.7	[36]
CdHgTe QDs@NaCl	80–340	1.4	[37]
CdTe QDs@NaCl	80–360	0.61	[8]
CdTe QDs-Layered Double Hydroxides ultrathin films	23–80	1.47	[21]
CdTe QDs solution	23–80	0.57	[21]
CdTe QDs film	23–80	0.83	[21]

**Table 3:** Temperature dependence of the PL peak wavelength of the Ag@SiO_2_@CdS/ZnS composite nanoparticle film sensor.

	Quadratic Function Relation	Adj. R-Square
BEE	$W=442.53043+0.00955T+7.92497\times 10^{-5}T^{2}$	0.99926
SSE	$W=515.34914+0.28934T-4.20406\times 10^{-4}T^{2}$	0.99528

**Table 4:** Temperature dependence of the PL peak wavelength of the Ag@SiO_2_@CdS/ZnS composite nanoparticle film sensor.

	Linear or Cubic Function Relation	Adj. R-Square
BEE	$W=2.2886-0.02474T+1.1848\times 10^{-4}T^{2}-1.95224\times 10^{-7}T^{3}$	0.99921
SSE	W=1.34914−0.00414T	0.99776

## Data Availability

Not applicable.

## Supplementary Materials

The following supporting information can be downloaded at: https://www.mdpi.com/article/10.3390/nano12224019/s1, Figure S1: The atomic force microscopy (AFM) diagram of a representative Ag@SiO_2_@CdS/ZnS composite nanoparticle film; Figure S2: Temperature-dependent result of CdS/ZnS QDs film.
