# Supplementary material for: Eye-Resolvable Surface-Plasmon-Enhanced Fluorescence Temperature Sensor
Source: Nanomaterials (Basel). 2022 Nov 16;12(22):4019. doi: 10.3390/nano12224019 (PMC9695039; doi:10.3390/nano12224019)
Supplement: Supplementary file 1 [file nanomaterials-12-04019-s001.zip › nanomaterials-1995541-supplementary.pdf]

Supplementary Materials

List of SI:

Figure S1. The atomic force microscopy (AFM) diagram of a representative $Ag@SiO_2@CdS/ZnS$ composite nanoparticle film.

Figure S2. Temperature-dependent result of CdS/ZnS QDs film.

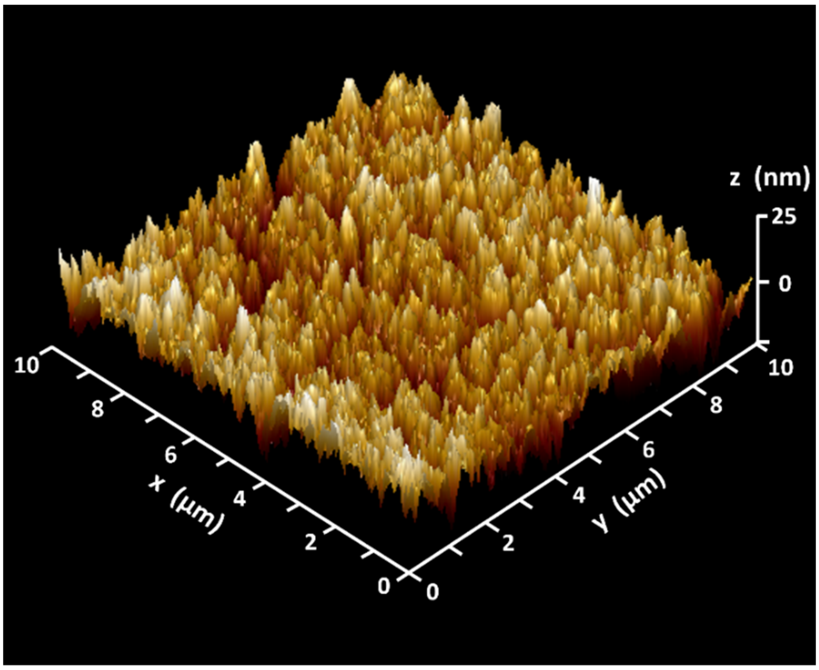

Figure S1. Morphology of an $Ag@SiO_2@CdS/ZnS$ composite nanoparticle film surface analyzed by atomic force microscopy (AFM).

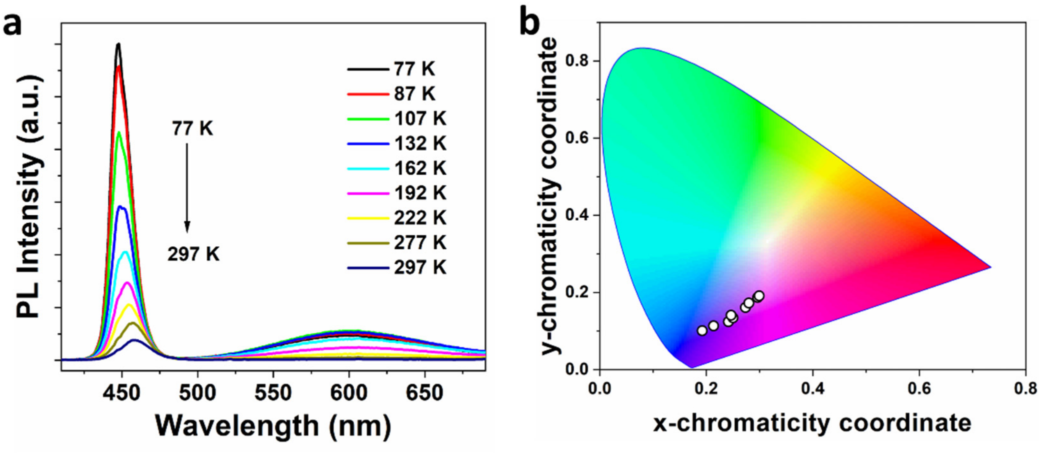

Figure S2. (a) The temperature-dependent PL spectra of CdS/ZnS QDs film in the 77–297 K temperature range, respectively. (b) Temperature-dependent emission from the CdS/ZnS QDs film projected onto the Commission Internationale del'Éclairage (CIE) chromaticity diagram based on PL spectra shown in Figure S2a.
